# Poly[dibromidobis[μ-1-(pyridin-4-ylmeth­yl)-1*H*-1,2,4-triazole-κ^2^ *N*:*N*′]cadmium]

**DOI:** 10.1107/S1600536811000535

**Published:** 2011-01-15

**Authors:** Xiu-Zhi Xu, Zhu-Lai Li, Wen-Jin Yan, Hui-Li Chi

**Affiliations:** a Department of Medicinal Chemistry, School of Pharmacy, Fujian Medical University, Fuzhou, Fujian 350004, People’s Republic of China

## Abstract

The title coordination polymer, [CdBr_2_(C_8_H_8_N_4_)_2_]_*n*_, arose from a layer-separated diffusion synthesis at room temperature. The title compound is isotypic with the I and Cl analogues. The Cd atom, located on an inversion center, is coordinated by two bromide ions and four N atoms (two from triazole rings and two from pyridyl rings) in a distorted *trans*-CdBr_2_N_4_ octa­hedral arrangement. The bridging 1-(4-pyridyl­meth­yl)-1*H*-1,2,4-triazole ligands are twisted [dihedral angle between the triazole and pyridine rings = 72.56 (13)°], affording a two-dimensional 4^4^ sheet structure in the crystal.

## Related literature

For structures of Cd(II) polymers with related ligands, see: Liu *et al.* (2005 ▶); Huang *et al.* (2006 ▶). For the structures of isotypic analogues with I and Cl, see: Wang *et al.* (2008 ▶, 2010 ▶). For the structure of the isotypic complex with Cu(II) and Cl, see: Li *et al.* (2009 ▶).
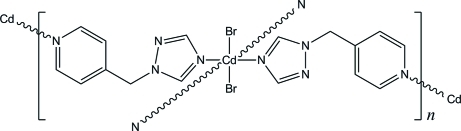


## Experimental

### 

#### Crystal data

[CdBr_2_(C_8_H_8_N_4_)_2_]
*M* *_r_* = 592.59
Monoclinic, 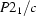

*a* = 7.7802 (9) Å
*b* = 16.7299 (16) Å
*c* = 8.4684 (10) Å
β = 114.409 (5)°
*V* = 1003.74 (19) Å^3^
*Z* = 2
Mo *K*α radiation
μ = 5.09 mm^−1^
*T* = 293 K
0.65 × 0.60 × 0.55 mm

#### Data collection

Rigaku Mercury70 CCD diffractometer
Absorption correction: multi-scan (*ABSCOR*; Higashi, 1995 ▶) *T*_min_ = 0.498, *T*_max_ = 1.000
7206 measured reflections
2270 independent reflections
2098 reflections with *I* > 2σ(*I*)
*R*_int_ = 0.022

#### Refinement

*R*[*F* ^2^ > 2σ(*F* ^2^)] = 0.024
*wR*(*F* ^2^) = 0.069
*S* = 0.91
2270 reflections
124 parameters
H-atom parameters constrained
Δρ_max_ = 0.74 e Å^−3^
Δρ_min_ = −0.51 e Å^−3^

### 

Data collection: *CrystalClear* (Rigaku/MSC, 2004 ▶); cell refinement: *CrystalClear*; data reduction: *CrystalClear*; program(s) used to solve structure: *SHELXS97* (Sheldrick, 2008 ▶); program(s) used to refine structure: *SHELXL97* (Sheldrick, 2008 ▶); molecular graphics: *SHELXTL* (Sheldrick, 2008 ▶); software used to prepare material for publication: *SHELXTL*.

## Supplementary Material

Crystal structure: contains datablocks I, global. DOI: 10.1107/S1600536811000535/bh2328sup1.cif

Structure factors: contains datablocks I. DOI: 10.1107/S1600536811000535/bh2328Isup2.hkl

Additional supplementary materials:  crystallographic information; 3D view; checkCIF report

## Figures and Tables

**Fig. 1.**
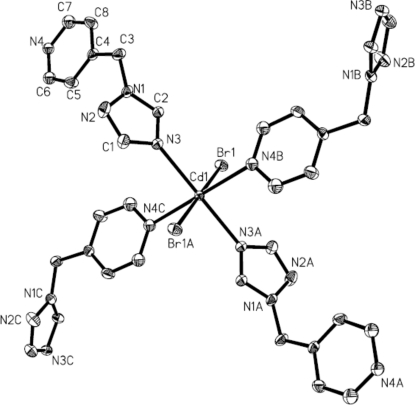
A view of the structure of the title compound, showing 30% probability displacement ellipsoids. H atoms have been omitted for clarity. Symmetry codes: (A) -x, -y + 1, -z; (B) -x + 1, y + 1/2, -z + 1/2; (C) x - 1, -y + 3/2, z - 1/2.

**Fig. 2.**
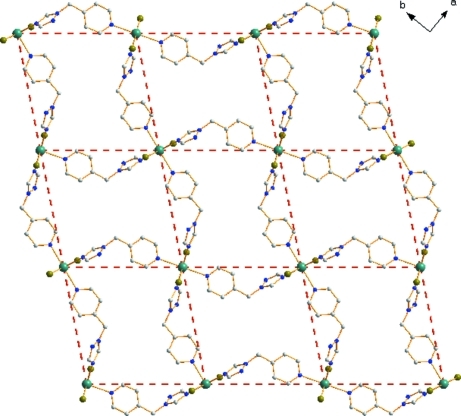
The two-dimensional structure of the title compound, constructed of rhombus-shaped grids.

## supplementary crystallographic information

### Comment

Recently, our group has focused on the design and synthesis of some flexible unsymmetric ligands (Liu *et al.*, 2005; Huang *et al.*, 2006), one of which being the heterocyclic ligand pyta, *N*-(4-pyridylmethyl)-1,2,4-triazole. In order to explore the architectural styles and other features of this kind of ligands, we selected cadmium dibromide as a representative subject for stereoregular coordination. Among our attempts, a new polymer [CdBr_2_(pyta)_2_]*_n_* was obtained as crystals suitable for single-crystal X-ray analysis.

The crystal structure of the title compound is isomorphous to other complexes we have reported with I or Cl in place of Br (Wang *et al.*, 2008, 2010) or with Cu(II) and Cl (Li *et al.*, 2009). The crystallographic analysis reveals that the title compound crystallizes in the monoclinic space group *P*2_1_/*c*. The asymmetric unit contains one cadmium atom, one bromide donor and one pyta bridging molecule, as shown in Fig. 1. The Cd(II) ion is placed on an inversion center, with an octahedral [CdBr_2_N_4_] environment, where the axial positions are occupied by two bromide ions and the equatorial positions occupied by two *trans* triazole N atoms and two *trans* pyridyl N atoms, each of which respectively belonging to four symmetry-related pyta ligands (Fig. 1). The bond angles about the Cd octahedron range from 85.88 (8) to 94.12 (8)° and deviate slightly from those of a perfect octahedron. Due to the existence of the —CH_2_— spacer between the triazole and the pyridyl ring, sufficient flexibility makes possible for pyta to be twisted in order to meet the requirement of coordination geometry of the metal center. The dihedral angle between the triazole and pyridyl rings in the ligand is 72.56 (13)°.

As conveniently shown in Fig. 2, the title compound forms an infinite two-dimensional rhombohedral sheet containing 36-membered sandglass rings. The *sp*^3^ hybridization of C3 forces the pyta ligand to be non-linear, generating the nonlinear grid sides and thereby the sandglass grids. Every complementary four [Cd_4_(pyta)_4_] grids are connected together by sharing the cadmium apices to give the 4^4^ two-dimensional structure with a side length of 11.01 Å, and a diagonal measurement of about 14.31 × 16.73 Å^2^.

### Experimental

A solution of pyta (0.021 g, 0.10 mmol) in MeOH (5 ml) was carefully layered on a solution of CdBr_2_ (0.027 g, 0.10 mmol) in H_2_O (5 ml). Diffusion between the two phases over a period of two weeks produced colorless block crystals.

### Refinement

All H atoms were placed in calculated positions and refined using a riding model with C—H bond lengths fixed to 0.93 (aromatic) or 0.97 Å (methylene), and isotropic displacement parameters calculated as 1.2 times the equivalent displacement parameter of the carrier C atom.

### Crystal data

**Table:**

[CdBr_2_(C_8_H_8_N_4_)_2_]	*F*(000) = 572
*M**_r_* = 592.59	*D*_x_ = 1.961 Mg m^−^^3^
Monoclinic, *P*2_1_/*c*	Mo *K*α radiation, λ = 0.71073 Å
Hall symbol: -P 2ybc	Cell parameters from 2944 reflections
*a* = 7.7802 (9) Å	θ = 2.6–27.5°
*b* = 16.7299 (16) Å	µ = 5.09 mm^−^^1^
*c* = 8.4684 (10) Å	*T* = 293 K
β = 114.409 (5)°	Block, yellow
*V* = 1003.74 (19) Å^3^	0.65 × 0.60 × 0.55 mm
*Z* = 2	

### Data collection

**Table:**

Rigaku Mercury70 CCD diffractometer	2270 independent reflections
Radiation source: fine-focus sealed tube	2098 reflections with *I* > 2σ(*I*)
graphite	*R*_int_ = 0.022
Detector resolution: 14.6306 pixels mm^-1^	θ_max_ = 27.5°, θ_min_ = 3.1°
ω scans	*h* = −10→10
Absorption correction: multi-scan (*ABSCOR*; Higashi, 1995)	*k* = −21→19
*T*_min_ = 0.498, *T*_max_ = 1.000	*l* = −10→10
7206 measured reflections	

### Refinement

**Table:**

Refinement on *F*^2^	Primary atom site location: structure-invariant direct methods
Least-squares matrix: full	Secondary atom site location: difference Fourier map
*R*[*F*^2^ > 2σ(*F*^2^)] = 0.024	Hydrogen site location: inferred from neighbouring sites
*wR*(*F*^2^) = 0.069	H-atom parameters constrained
*S* = 0.91	*w* = 1/[σ^2^(*F*_o_^2^) + (0.048*P*)^2^ + 0.7729*P*] where *P* = (*F*_o_^2^ + 2*F*_c_^2^)/3
2270 reflections	(Δ/σ)_max_ = 0.083
124 parameters	Δρ_max_ = 0.74 e Å^−^^3^
0 restraints	Δρ_min_ = −0.51 e Å^−^^3^
0 constraints	

### Fractional atomic coordinates and isotropic or equivalent isotropic displacement parameters (Å^2^)

**Table:**

	*x*	*y*	*z*	*U*_iso_*/*U*_eq_	
Cd1	0.0000	0.5000	0.5000	0.02280 (9)	
Br1	0.19066 (4)	0.538566 (18)	0.30452 (4)	0.03501 (10)	
C1	0.2617 (4)	0.57653 (19)	0.8969 (4)	0.0371 (6)	
H1A	0.1582	0.5731	0.9248	0.044*	
C2	0.4305 (4)	0.56459 (17)	0.7599 (4)	0.0303 (5)	
H2A	0.4757	0.5526	0.6768	0.036*	
C3	0.7295 (4)	0.61998 (17)	0.9870 (4)	0.0380 (7)	
H3A	0.7919	0.5889	0.9293	0.046*	
H3B	0.7836	0.6045	1.1082	0.046*	
C4	0.7711 (4)	0.70758 (15)	0.9750 (4)	0.0297 (6)	
C5	0.6428 (4)	0.76858 (18)	0.9397 (5)	0.0404 (7)	
H5A	0.5176	0.7576	0.9173	0.048*	
C6	0.7013 (4)	0.84676 (17)	0.9379 (4)	0.0388 (7)	
H6A	0.6129	0.8875	0.9146	0.047*	
C7	1.0005 (4)	0.8062 (2)	1.0007 (5)	0.0458 (8)	
H7A	1.1243	0.8186	1.0204	0.055*	
C8	0.9543 (5)	0.72732 (18)	1.0071 (5)	0.0439 (8)	
H8A	1.0458	0.6877	1.0329	0.053*	
N1	0.5308 (3)	0.59953 (13)	0.9121 (3)	0.0299 (5)	
N2	0.4232 (4)	0.60782 (17)	1.0010 (3)	0.0421 (6)	
N3	0.2588 (3)	0.54957 (14)	0.7446 (3)	0.0293 (5)	
N4	0.8769 (3)	0.86608 (13)	0.9675 (3)	0.0327 (5)	

### Atomic displacement parameters (Å^2^)

**Table:**

	*U*^11^	*U*^22^	*U*^33^	*U*^12^	*U*^13^	*U*^23^
Cd1	0.02036 (14)	0.01935 (14)	0.02797 (15)	0.00065 (9)	0.00926 (11)	−0.00225 (9)
Br1	0.03130 (17)	0.04281 (18)	0.03652 (17)	0.00314 (12)	0.01962 (13)	0.00383 (11)
C1	0.0407 (16)	0.0381 (15)	0.0380 (15)	−0.0095 (13)	0.0219 (13)	−0.0070 (12)
C2	0.0263 (13)	0.0304 (13)	0.0339 (14)	−0.0032 (11)	0.0120 (11)	−0.0059 (11)
C3	0.0299 (14)	0.0247 (13)	0.0469 (17)	−0.0070 (11)	0.0033 (13)	−0.0006 (12)
C4	0.0316 (14)	0.0218 (12)	0.0318 (13)	−0.0059 (10)	0.0092 (11)	−0.0013 (10)
C5	0.0278 (14)	0.0297 (14)	0.0595 (19)	−0.0051 (11)	0.0138 (14)	0.0007 (13)
C6	0.0298 (14)	0.0243 (13)	0.0595 (19)	0.0013 (11)	0.0157 (14)	0.0046 (12)
C7	0.0329 (16)	0.0255 (15)	0.083 (3)	−0.0035 (12)	0.0282 (17)	−0.0032 (14)
C8	0.0340 (15)	0.0234 (14)	0.075 (2)	−0.0006 (12)	0.0227 (16)	−0.0022 (13)
N1	0.0309 (12)	0.0221 (10)	0.0320 (11)	−0.0059 (9)	0.0083 (10)	−0.0018 (8)
N2	0.0496 (16)	0.0464 (15)	0.0362 (13)	−0.0175 (13)	0.0235 (12)	−0.0127 (11)
N3	0.0257 (11)	0.0310 (12)	0.0297 (11)	−0.0026 (9)	0.0100 (9)	−0.0046 (9)
N4	0.0328 (12)	0.0221 (11)	0.0449 (14)	−0.0022 (9)	0.0177 (11)	0.0007 (9)

### Geometric parameters (Å, °)

**Table:**

Cd1—N3	2.363 (2)	C3—H3A	0.9700
Cd1—N3^i^	2.363 (2)	C3—H3B	0.9700
Cd1—N4^ii^	2.407 (2)	C4—C5	1.372 (4)
Cd1—N4^iii^	2.407 (2)	C4—C8	1.376 (4)
Cd1—Br1	2.7178 (4)	C5—C6	1.387 (4)
Cd1—Br1^i^	2.7178 (3)	C5—H5A	0.9300
C1—N2	1.310 (4)	C6—N4	1.323 (4)
C1—N3	1.358 (4)	C6—H6A	0.9300
C1—H1A	0.9300	C7—N4	1.335 (4)
C2—N3	1.312 (4)	C7—C8	1.375 (4)
C2—N1	1.334 (4)	C7—H7A	0.9300
C2—H2A	0.9300	C8—H8A	0.9300
C3—N1	1.449 (4)	N1—N2	1.345 (4)
C3—C4	1.513 (4)	N4—Cd1^iv^	2.407 (2)
			
N3—Cd1—N3^i^	180.0	H3A—C3—H3B	107.6
N3—Cd1—N4^ii^	85.88 (8)	C5—C4—C8	117.8 (3)
N3^i^—Cd1—N4^ii^	94.12 (8)	C5—C4—C3	125.3 (3)
N3—Cd1—N4^iii^	94.12 (8)	C8—C4—C3	116.9 (3)
N3^i^—Cd1—N4^iii^	85.88 (8)	C4—C5—C6	119.4 (3)
N4^ii^—Cd1—N4^iii^	180.0	C4—C5—H5A	120.3
N3—Cd1—Br1	88.21 (6)	C6—C5—H5A	120.3
N3^i^—Cd1—Br1	91.79 (6)	N4—C6—C5	123.1 (3)
N4^ii^—Cd1—Br1	90.06 (6)	N4—C6—H6A	118.4
N4^iii^—Cd1—Br1	89.94 (6)	C5—C6—H6A	118.4
N3—Cd1—Br1^i^	91.79 (6)	N4—C7—C8	123.4 (3)
N3^i^—Cd1—Br1^i^	88.21 (6)	N4—C7—H7A	118.3
N4^ii^—Cd1—Br1^i^	89.94 (6)	C8—C7—H7A	118.3
N4^iii^—Cd1—Br1^i^	90.06 (6)	C7—C8—C4	119.3 (3)
Br1—Cd1—Br1^i^	180.0	C7—C8—H8A	120.4
N2—C1—N3	114.0 (3)	C4—C8—H8A	120.4
N2—C1—H1A	123.0	C2—N1—N2	109.6 (2)
N3—C1—H1A	123.0	C2—N1—C3	128.3 (3)
N3—C2—N1	110.2 (3)	N2—N1—C3	121.9 (2)
N3—C2—H2A	124.9	C1—N2—N1	103.2 (2)
N1—C2—H2A	124.9	C2—N3—C1	103.0 (2)
N1—C3—C4	114.7 (2)	C2—N3—Cd1	128.17 (19)
N1—C3—H3A	108.6	C1—N3—Cd1	128.52 (19)
C4—C3—H3A	108.6	C6—N4—C7	117.0 (2)
N1—C3—H3B	108.6	C6—N4—Cd1^iv^	125.46 (19)
C4—C3—H3B	108.6	C7—N4—Cd1^iv^	117.12 (19)

Symmetry codes: (i) −*x*, −*y*+1, −*z*+1; (ii) *x*−1, −*y*+3/2, *z*−1/2; (iii) −*x*+1, *y*−1/2, −*z*+3/2; (iv) −*x*+1, *y*+1/2, −*z*+3/2.
